# A Myeloid-Specific Lack of IL-4Rα Prevents the Development of Alternatively Activated Macrophages and Enhances Immunity to Experimental Cysticercosis

**DOI:** 10.3390/pathogens13020169

**Published:** 2024-02-13

**Authors:** Jonadab E. Olguín, Edmundo Corano-Arredondo, Victoria Hernández-Gómez, Irma Rivera-Montoya, Mario A. Rodríguez, Itzel Medina-Andrade, Berenice Arendse, Frank Brombacher, Luis I. Terrazas

**Affiliations:** 1 Laboratorio Nacional en Salud: Diagnóstico Molecular y Efecto Ambiental en Enfermedades Crónico-Degenerativas, Facultad de Estudios Superiores Iztacala, Universidad Nacional Autónoma de México (UNAM), Tlalnepantla 54090, Estado de México, Mexico; je.olguin@iztacala.unam.mx (J.E.O.);; 2 Unidad de Biomedicina, Facultad de Estudios Superiores Iztacala, UNAM, Tlalnepantla 54090, Estado de México, Mexico; 3 Center for Infectious Medicine (CIM), Department of Medicine, Hudinge, Karolinska Institutet, 141 52 Stockholm, Sweden; 4 Institute of Infectious Diseases and Molecular Medicine, University of Cape Town, Cape Town 7925, South Africa

**Keywords:** helminth infection, alternatively activated macrophages, *Taenia crassiceps*

## Abstract

To determine the role that the IL-4/IL13 receptor plays in the development of alternatively activated macrophages (AAM or M2) and their role in the regulation of immunity to the extraintestinal phase of the helminth parasite *Taenia crassiceps*, we followed the infection in a mouse strain lacking the IL-4Rα gene (IL-4Rα^−/−^) and in the macrophage/neutrophil-specific IL-4Rα-deficient mouse strain (LysMcreIL-4Rα^−/lox^ or cre/LoxP). While 100% of *T. crassiceps*-infected IL-4Rα^+/+^ (WT) mice harbored large parasite loads, more than 50% of th eIL-4Rα^−/−^ mice resolved the infection. Approximately 88% of the LysMcreIL-4Rα^−/lox^ mice displayed a sterilizing immunity to the infection. The remaining few infected cre/LoxP mice displayed the lowest number of larvae in their peritoneal cavity. The inability of the WT mice to control the infection was associated with antigen-specific Th2-type responses with higher levels of IgG1, IL-4, IL-13, and total IgE, reduced NO production, and increased arginase activity. In contrast, IL-4Rα^−/−^ semi-resistant mice showed a Th1/Th2 combined response. Furthermore, macrophages from the WT mice displayed higher transcripts for Arginase-1 and RELM-α, as well as increased expression of PD-L2 with robust suppressive activity over anti-CD3/CD28 stimulated T cells; all of these features are associated with the AAM or M2 macrophage phenotype. In contrast, both the IL-4Rα^−/−^ and LysMcreIL-4Rα^−/lox^ mice did not fully develop AAM or display suppressive activity over CD3/CD28 stimulated T cells, reducing PDL2 expression. Additionally, T-CD8^+^ but no T-CD4^+^ cells showed a suppressive phenotype with increased Tim-3 and PD1 expression in WT and IL-4Rα^−/−^, which were absent in *T. crassiceps*-infected LysMcreIL-4Rα^−/lox^ mice. These findings demonstrate a critical role for the IL-4 signaling pathway in sustaining AAM and its suppressive activity during cysticercosis, suggesting a pivotal role for AAM in favoring susceptibility to *T. crassiceps* infection. Thus, the absence of these suppressor cells is one of the leading mechanisms to control experimental cysticercosis successfully.

## 1. Introduction

Helminth infections are widely known to lead to T helper type 2 (Th2) responses typified by high levels of the profile of cytokines they induce, such as IL-4, IL-5, IL-10, and IL-13, accompanied by high levels of characteristic antibodies such as IgG1 and IgE, as well as increased numbers of eosinophils, goblet cells, and mast cells [1]. Moreover, impaired T cell proliferative responses to polyclonal stimuli, specific parasite antigens, and “bystander” antigens are also observed. This type of response is frequently assumed to mediate resistance to most helminth infections [2].

Macrophages play crucial roles in the immune response as they can initiate, modulate, and be the final effector cells during responses to infections. These cells are classified in classically (M1) and alternatively (M2) activated macrophages. M1 macrophages are involved in the production of IL-6, IL-12, and TNF-α after LPS and IFN-γ activation, increasing the expression of MHCII, CD80, and CD86 surface molecules [3], with them having essential roles in immunity against intracellular parasites through the production of high levels of reactive oxygen and nitrogen intermediates such as nitric oxide (NO) and reactive oxygen species (ROS) [4]. On the other hand, M2 macrophages are involved in tissue repair and the anti-inflammatory response through IL-10 production and the expression of Arginase-1 and Resistin-like molecule (RELM)-α [3,5,6]. Here, the activation of the L-arginine amino acid pathway plays a critical role and determines the bias mechanism of macrophages, where activation by IFN-γ and TNF-α in M1 macrophages favors nitric oxide (NO) production by increasing nitric-oxide synthase 2 (NOS2) activity. In contrast, increased arginase-1 (Arg-1) activity is observed in M2 in response to IL-4 and IL-13, favoring L-arginine metabolism towards proline, polyamine, and urea production [7].

Interleukin-4 is a pleiotropic cytokine secreted by activated T cells, mast cells, basophils, and eosinophils [8,9]. Likewise, high-affinity IL-4 receptors are expressed on various cell types, including hematopoietic, endothelial, epithelial, and fibroblast cells [9]. IL-4 signalizes through the 140-kDa high-affinity IL-4 receptor α chain (IL-4Rα) associated with the γ common subunit (γC), generating the IL-4/IL-4Rα-complex. The IL-4Rα chain also associates with the IL-13Rα1 chain to form a high-affinity IL-13 receptor. Thus, the IL-4Rα chain is critical for the biological activities of IL-4 and IL-13, inducing STAT6-mediated signaling [10].

As much as other classes of helminths, *Taenia crassiceps* infection triggers polarized Th2-type responses, inducing chronic infection. Likewise, we and others isolated M2 macrophages from *T. crassiceps*-infected mice with the ability to inhibit naïve T cell responses [11,12]. This effect involves a cell contact-dependent pathway mediated by the interaction between PD-1 and its ligands [12]. Hence, we are interested in demonstrating the influence of IL-4Rα in developing and maintaining alternatively activated macrophages (AAMs or M2) and its role in the outcome of murine cysticercosis caused by the cestode *T. crassiceps*. To address this question, we followed the course of *T. crassiceps* infection in LysMcreIL-4Rα^−/lox^ (cre/LoxP) mice, which specifically have an absence of IL-4Rα in their macrophages, while other cell types maintain its expression [13] compared with infected WT and IL-4Rα^−/−^ BALB/c mice. Our data have revealed a crucial role for IL-4Rα in developing susceptibility during murine cysticercosis, which is associated with M2 macrophages.

## 2. Materials and Methods

Mice. Six- to eight-week-old female BALB/c, IL-4Rα^−/−^ and LysMcreIL-4Rα^−/lox^ (cre/LoxP) mice were maintained in the animal facility Facultad de Estudios Superiores Iztacala according to institutional and national guidelines [14]. Both the IL-4Rα-KO mice and the LysMcreIL-4Rα^−/lox^ mice were donated by Dr. F. Bromabacher (Cape Town, SA).

Parasites and infection. Metacestodes of *Taenia crassiceps* (ORF strain) were harvested in sterile conditions from the peritoneal cavity of female BALB/c mice after 8–10 weeks of infection. The cysticerci were washed twice in phosphate-buffered saline (PBS; 0.15 M NaCl, 0.01 M sodium phosphate buffer, pH 7.2) and used for mouse infection. The mice were infected with an intraperitoneal (i.p.) injection of 10 cysticerci of *T. crassiceps*. The infected mice were sacrificed at weeks 2, 4, and 8 post-infection (wpi), and the parasite number was obtained.

Antibody ELISA. Peripheral blood was collected 2, 4, and 8 wpi following tail snips of the *T. crassiceps*-infected mice. *T. crassiceps-specific* IgG1 and IgG2a levels were determined by ELISA, as previously described [15]. The results are expressed as the maximum serum dilution of antibody detection. Total IgE production was detected using the Opt-ELISA (Biolegend). According to the manufacturer’s instructions, IL-4 and IL-13 levels were measured using an ELISA sandwich (Peprotech-México, México City, Mexico).

Cell culture for cytokine production. Spleens from the infected mice were removed in sterile conditions. Single-cell suspensions were cultured in RPMI-1640 supplemented with 10% fetal bovine serum, 100 units of penicillin/streptomycin, two mM glutamine, 25 mM HEPES buffer, and 1% non-essential amino acids (all from GIBCO) (ThermoFisher, Mexico City, Mexico). The cells were centrifuged, and the erythrocytes were lysed by resuspending the cells in lysis solution (39 mM NH_4_Cl, 2.5 mM KHCO_3_, and 0.02 mM EDTA2.Na). Following two washes, the viable cells were counted through the use of trypan blue exclusion with a Countess II FL system (Applied Biosystems, Waltham, MA, USA). The splenocytes were adjusted to 3 × 10^6^ cells/mL in the same medium. Next, 3 × 10^5^ cells were cultured in 100 µL into 96-well flat bottom culture plates (Costar, Cambridge, MA, USA) stimulated with either plate-bound anti-CD3 antibody (1 μg/mL) or a soluble extract of *T. crassiceps* (25 μg/mL) at 37 °C for 72 or 96 h, respectively. IL-4 and IFN-γ were measured using a sandwich ELISA according to the manufacturer’s instructions (Peprotech-México, México City, Mexico). IL-4, IL-6, IFN-γ, and TNF-α in the supernatant were evaluated using a CBA Mouse Inflammation Kit (BD Biosciences, Mexico City, Mexico) according to the manufacturer’s instructions.

Isolation and activation of peritoneal macrophages. Peritoneal exudate cells (PECs) were obtained from the peritoneal cavity of 8-week-old *T. crassiceps*-infected mice. The cells were washed twice with a cold saline solution. Viable cells were counted through the use of trypan blue exclusion in Countess II FL (Applied Biosystem). PECs were adjusted to 5 × 10^6^ cells/mL in supplemented RPMI and cultured in 6-well plates (Costar) for 2 h at 37 °C and 5% CO_2_. Non-adherent cells were removed by washing them with a warm supplemented RPMI medium. The adherent cells were removed using EDTA and readjusted to 1 × 10^6^ cells/mL. Their viability was rechecked at this point (>90%). The cells (1 mL) were incubated in 24-well plates at 37 °C and 5% CO_2_ for 24 h. Macrophages were harvested for classical or activated gene expression using RT-PCR assays. The cells were >90% macrophage positive to CD11b and F4/80 surface markers, as determined by flow cytometry. Supernatants were used for arginase and nitric oxide activity.

RT-PCR assay. The expression of arginase 1 (*ARG-1*), inducible nitric oxide synthase (*INOS*), interleukin-4 receptor alpha (*IL4RA*), and Resistin-like molecule-α (*RELMA*) mRNA transcripts in peritoneal macrophages was determined by reverse transcription (RT)-PCR. Adherent peritoneal macrophages from *T. crassiceps*-infected BALB/c mice were aseptically removed at the indicated time points. TRIzol reagent (Invitrogen, Carlsbad, CA, USA) was used for RNA extraction using the propanol–chloroform technique. The RNA was quantified, and 3 μg of RNA was reverse transcribed using a Superscript II First Strand Synthesis Kit (Invitrogen, Waltham, MA, USA) and an oligo dT primer. Once cDNA was obtained, conventional PCR was performed as previously described [14]. Finally, a 1.5% agarose gel was prepared to observe the amplified products, with the samples being loaded with blue juice buffer containing SYBR Green (Invitrogen). Visualization of gels was performed with a Fujifilm FLA 5000 scanner (Fuji, Tokyo, Japan) with FLA 5000 image reader V2.1 software.

Evaluation of nitric oxide production and arginase activity. The nitric oxide level in the supernatant of the macrophages cultured was determined by the increase in nitrite concentration [16] using the Griess reaction in the microwell plates (Costar), as previously described [17]. Arginase activity was determined with an Arginase Activity Assay Kit (Sigma-Aldrich, Saint Louis, MO, USA) according to the manufacturer’s instructions, where arginase catalyzes the conversion of arginine to urea and ornithine.

Flow cytometry. Once the peritoneal cells were adjusted (1 × 10^6^ cells/mL), the Fc receptors were blocked with anti-mouse CD16/CD32 (Biolegend, San Diego, CA, USA) for staining with Ly6C FITC, Ly6G APC/Cy7, CD11b PE/Cy5.5, F4/80 APC, MHCII APC/Fire, PDL2 PE, CD4 BV421, CD8 BV605, Tim-3 APC, PD1 PE and CD25 BV711 (all from Biolegend, San Diego, CA, USA). sAfter being washed, the cells were resuspended and analyzed in either FACSAria Fusion (BD Biosciences, Mexico City, Mexico) or Attune NxT (ThermoFisher, Waltham, MA, USA) from Laboratorio Nacional en Salud Core Facility Area, FES Iztacala. Data were analyzed using FlowJo software V10.6 (BD Biosciences).

Suppression assays. A co-culture of macrophages obtained from infected mice with naive CD90 cells was performed as previously reported [12]. Splenocytes were enriched for CD90+ cells (95% according to FACS analysis) using magnetic cell sorter beads (MACS, Miltenyi Biotec, Bergisch Gladbach, Germany). The CD90 cells were plated in 96 well-flat bottom plates with 1 μg/mL of anti-CD3 and anti-CD28 antibodies (BioLegend, San Diego, CA, USA). Three hours later, macrophages were added to the CD90 T cells at ratios of 1:4, 1:8, and 1:16 (macrophages: CD90 T cells) maintained at 37 °C and 5% CO_2_ for 72 h for the [3H] thymidine incorporation assay (Amersham, England); the cells were incubated for 18 h with 0.5 μCi/well until harvested (Tomtec, Munich, Germany) and then, they were counted using a 1450 microplate counter (Mustionkatu, Turku, Finland). The values are represented as counts per minute (CPM) from triplicate wells.

Statistical analysis. The group comparisons were performed using one-way ANOVA with Tukey’s multiple comparisons test. Analyses were determined using Graph Pad Prism 7 software (Graph Pad, San Diego, CA, USA).

## 3. Results

### 3.1. The Absence of IL-4Rα Favors Resistance to Cysticercosis

**Figure 1:**
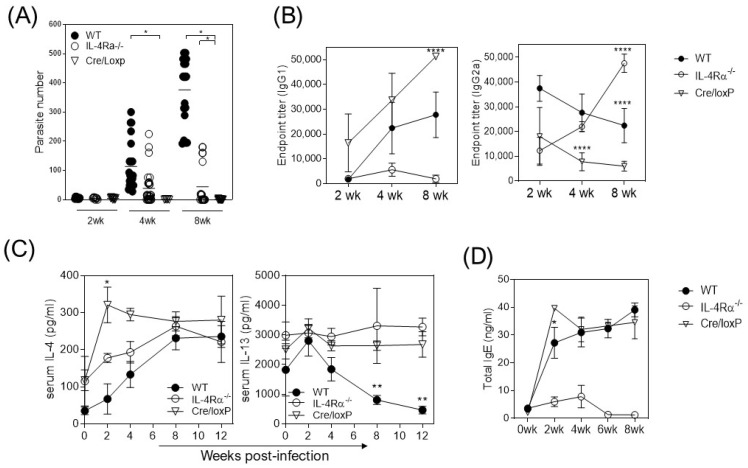
The absence of IL-4Rα signaling in macrophages favors resistance to *T. crassiceps* but does not change IL-4 production. The mice were infected with ten parasites and euthanized at marked times. (**A**) Parasite load in the peritoneal cavity at two, four, and eight weeks after infection in the WT, IL-4Rα^−/−^, and Cre/LoxP mouse strains. (**B**) Antigen-specific titers of IgG1 and IgG2a. (**C**) Detection of IL-4 and IL-13 in sera, and (**D**) IgE quantification in serum samples of infected mice from the three groups described at two, four, and eight weeks post-infection. Total data from 3 different experiments. Statistical differences were examined using one-way ANOVA with Tukey’s multiple comparisons post-test, considering significant a * *p* < 0.05, ** *p* < 0.01, and **** *p* < 0.0001.

It is widely accepted that IL-4-dependent type 2 immunity is critical in mediating protection against helminths by activating eosinophils, ILC2, and M2 macrophages [18]. However, during cysticercosis caused by *T. crassiceps* infection, we observed an increased number of parasites in the peritoneal cavity of WT mice at four and eight weeks post-infection (wpi, Figure 1A) which was associated with a Th2-biased profile in the antibody response, with increased IgG1 and total IgE and reduced IgG2a production (Figure 1B) and IL-4 but not IL-13 detection in serum (Figure 1C). In the absence of whole IL-4Rα signaling, *T. crasssiceps*-infected IL-4Rα^−/−^ mice displayed a reduced number of cysticerci in their peritoneal cavities, with the parasite load being reduced by about 50% compared to the WT mice (Figure 1A). We also detected a Th1-associated IgG2a subtype of antibodies as well as impaired IgG1 and IgE production (Figure 1B). Interestingly, the LysMcreIL-4Rα^−/lox^ (cre/LoxP) mice, whose macrophages have a lineage-specific IL-4Rα absence, showed low or no parasite load, with the infection being resolved in 88% of the mice as early as four weeks after infection (Figure 1A). These *T. crassiceps*-infected cre/LoxP mice displayed higher IgG1 antibody production at eight wpi and increased total IgE antibody production but reduced IgG2a titers and increased levels of IL-4 and IL-13 cytokines in serum (Figure 1A–C). These findings suggest that the IL-4Rα-mediated pathway in macrophages is involved in the pathogenesis of *T. crassiceps* infection in susceptible BALB/c mice.

### 3.2. The Absence of Total IL-4Rα or the Myeloid Lineage-Specific Absence of IL-4Rα Favors IFN-γ and TNF-α Production in T. crassiceps-Infected Mice

**Figure 2:**
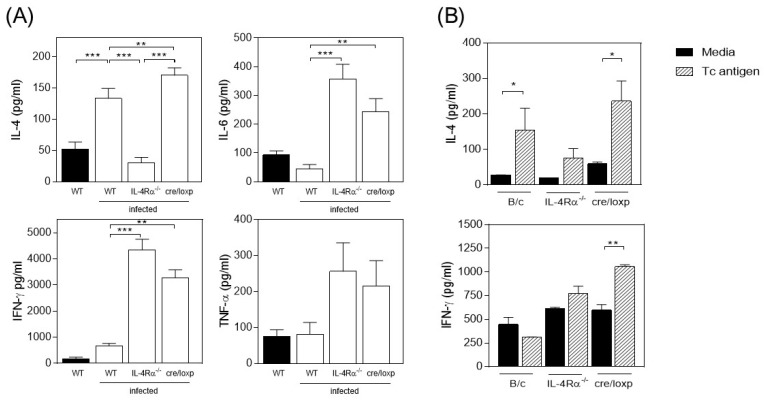
Dual Th1 and Th2 cytokine production in the myeloid-specific absence of IL-4Rα signaling in macrophages during *T. crassiceps* infection. Splenocytes from the different groups described were stimulated with (**A**) anti-CD3/CD28 antibodies for 72 h or (**B**) *T. crassiceps* soluble extract for 96 hrs. Cytokines were evaluated using ELISA or CBA as described in the Materials and Methods. Total data are from 3 different experiments. Statistical differences were examined using one-way ANOVA with Tukey’s multiple comparisons post-test, considering significant a * *p* < 0.05, ** *p* < 0.01, and *** *p* < 0.001.

Next, at eight wpi, α-CD3/CD28 cultured splenocytes from infected mice were evaluated for both pro- and anti-inflammatory cytokine production. As expected, WT spleen cells showed increased IL-4 production and reduced IL-6, IFN-γ, and TNF-α production (Figure 2A). In the absence of IL-4Rα signaling, the cells switched their cytokine profile, showing reduced IL-4 production but increased production of IL-6, IFN-γ, and TNF-α (Figure 2A). In contrast, *T. crassiceps*-infected cre/LoxP mice showed a dual profile of cytokine production, with increased IL-4, IL-6, IFN-γ, and TNF-α production (Figure 2A). *T. crassiceps* antigen-specific stimulated splenocytes from the cre/LoxP mice showed increased production of both IL-4 and IFN-γ cytokines. In contrast, the WT mice produced higher levels of IL-4 but reduced IFN-γ production (Figure 2B).

### 3.3. T. crassiceps-Infected cre/loxP Mice Maintain a Pro-Inflammatory Profile despite the Chronicity of the Infection

**Figure 3:**
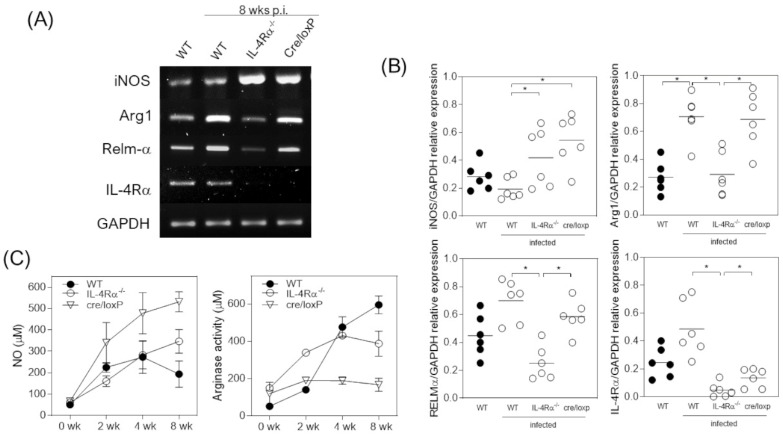
The myeloid-specific absence of IL-4Rα signaling induces a dual profile of macrophages. Cultured peritoneal exudate cells processed as described in the Materials and Methods were analyzed for *iNOS, ARG1, RELM-A, IL-4RA*, and *GAPDH* cDNA expression. (**A**) Representative and (**B**) total data. (**C**) Supernatants of cultured cells were analyzed for NO production and arginase activity. Total data from at least three different experiments. Statistical differences were examined using one-way ANOVA with Tukey’s multiple comparisons post-test, considering significant a * *p* < 0.05.

M2 macrophages play a significant role during helminth infections by encapsulating parasites and repairing tissues [19]. However, in the case of cysticercosis caused by *T. crassiceps* infection, protection is associated with the M1-STAT1-dependent profile of activation in macrophages [15]. To describe the gene expression profile of either M1 or M2 in peritoneal macrophages from 8 wpi mice, we evaluated induced nitrogen oxide synthase (iNOS) and IL-4Rα genes using RT-PCR. *T. crassiceps*-infected WT mice showed an M2 macrophage profile with increased IL-4Rα expression but reduced iNOS gene expression (Figure 3A,B). In the absence of IL-4Rα signaling, macrophages showed an increased M1 profile, with high iNOS expression and null IL-4Rα gene expression (Figure 3A,B). However, arginase activity assays and NO production in the supernatant of the cultured macrophages indicated an M1 profile in the cre/LoxP mice that may be associated with resistance (Figure 3C). These data strengthen the hypothesis that the dual activation profile of the immune response with pro- and anti-inflammatory characteristics is happening at the same time and is involved in protection during *T. crassiceps* infection, where macrophages play a significant role in inducing resistance to disease through NO production.

### 3.4. Cell Recruitment at the Site of Infection Is Altered in the Total or Cell Lineage-Specific Absence of IL-4Rα after T. crassiceps Infection

**Figure 4:**
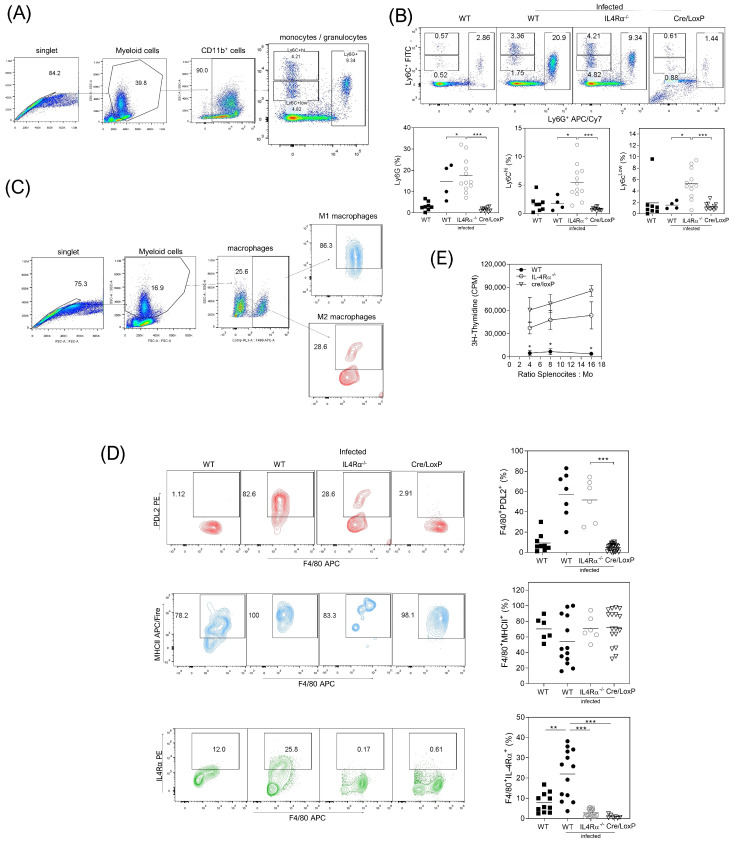
Reduced PDL2 percentage in the myeloid-specific absence of IL-4Rα signaling suggests an M1 profile during *T. crassiceps* infection. (**A**) Analysis strategy for (**B**) myeloid Ly6C and Ly6G cells in samples from the peritoneum in all of the experimental groups, with representative (upper) and total (lower) data from 3 different experiments. (**C**) Analysis strategy for (**D**) macrophages in samples from the peritoneum in all of the experimental groups, with representative (left) and total (right) data from 4 different experiments. (**E**) Co-culture of macrophages obtained from infected mice with naive CD90 cells as described in the Materials and Methods. Values are represented as counts per minute (CPM) from triplicate wells. Total data are from at least three different experiments. Statistical differences were examined using one-way ANOVA with Tukey’s multiple comparisons post-test, considering significant a * *p* < 0.05, ** *p* < 0.01 and *** *p* < 0.001.

After eight wpi, we analyzed CD11b^+^ myeloid cells from monocytic (Ly6C) and granulocytic (Ly6G) lineages recruited at the site of infection (peritoneum). Whilst these cells were almost absent in the naïve mice, the WT mice recruited increased percentages of granulocytic Ly6G and monocytic Ly6C^high^ cells after *T. crassiceps* infection (Figure 4B). In the absence of IL-4Rα signaling, *T. crassiceps*-infected mice showed higher recruitment of three myeloid lineages, Ly6G, Ly6C^low^, and Ly6C^high^ (Figure 4A,B). Interestingly, the *T. crassiceps*-infected cre/LoxP mice had no recruitment of these myeloid cell lineages at this time after infection. Additionally, peritoneal macrophages from the WT and IL4Rα^−/−^ infected mice displayed increased expression of PDL2 molecules, which was not observed in the cre/LoxP mice. In contrast, all mouse strains displayed high levels of MHCII expression (Figure 4C,D).

Next, suppression assays using peritoneal macrophages co-cultured with naive T cells stimulated with anti-CD3/CD28 antibodies confirmed that WT macrophages displayed strong suppressive activity, whereas IL-4Rα^−/−^ mice macrophages displayed medium suppressive activity (Figure 4E). In contrast, macrophages from *T. crassiceps*-infected cre/LoxP mice did not show suppression properties.

**Figure 5:**
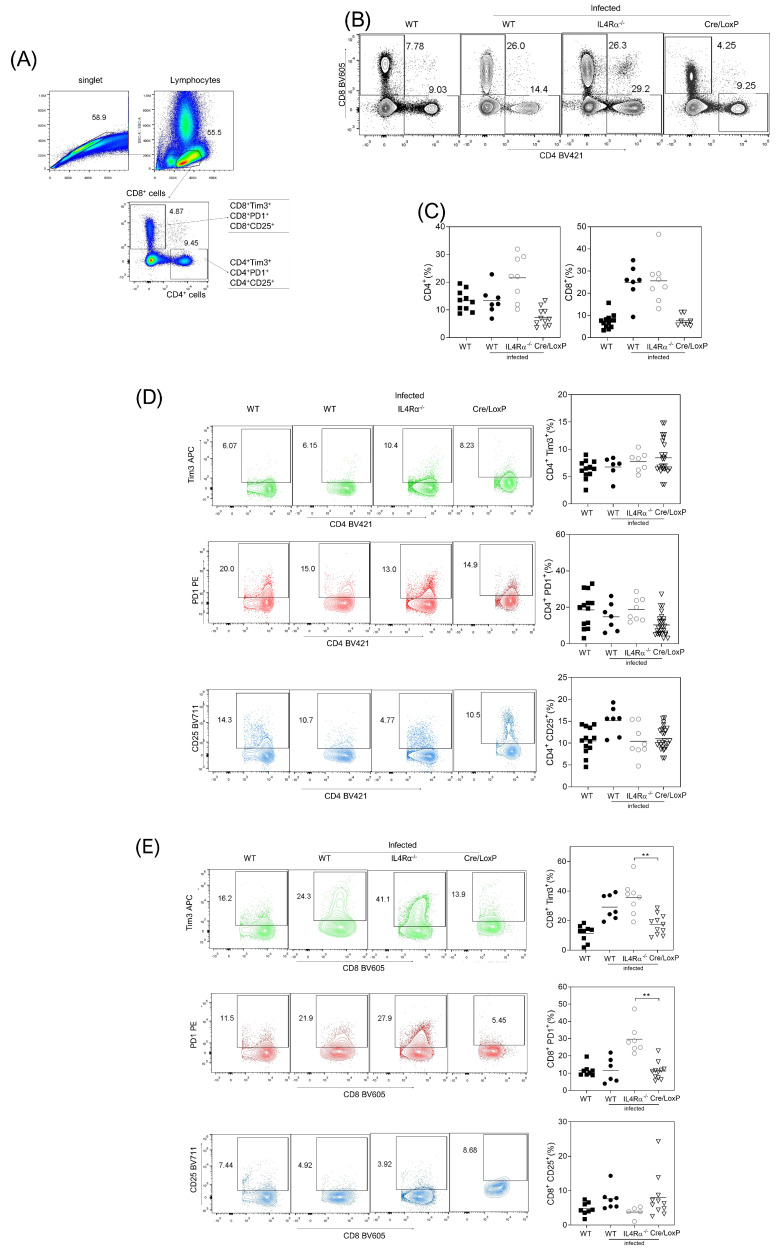
The suppressive profile in CD8+ cells is reduced in the myeloid-specific absence of IL-4Rα signaling during *T. crassiceps* infection. (**A**) Analysis strategy for CD4^+^ and CD8^+^ T cells in samples from the peritoneum in all of the experimental groups, with representative (**B**) and total (**C**) data of CD4^+^ and CD8^+^ cells, following the analyses for (**D**) Tim-3, PD1, and CD25 percentages in the CD4^+^ population or (**E**) Tim-3, PD1, and CD25 percentages in the CD8 population, with representative (left) or total (right) data from at least four different experiments. Statistical differences were examined using one-way ANOVA with Tukey’s multiple comparisons post-test, considering significant a ** *p* < 0.01.

Finally, we analyzed the profile of lymphoid T-CD4^+^ and T-CD8^+^ cells co-expressing either suppressor Tim-3 and PD1 or activated CD25 molecules at the site of infection at eight wpi. The CD4^+^ cells showed no differences between the groups (Figure 5A–D). However, the CD8^+^ cells showed increased Tim-3 and PD1 molecule expression in the WT and IL-4Rα^−/−^ mice, whereas the CD8^+^ cells from the cre/loxP mice displayed reduced expression of both suppressive markers (Figure 5E). Also, the rate of CD8^+^CD25^+^ cells increased in the cre/LoxP mice but did not reach significant differences. These data suggest that the suppressive profile is present in myeloid cells and T-CD8^+^ cells, specifically in WT and IL-4Rα^−/−^ infected mice.

## 4. Discussion

The immune response against parasite helminths, where myeloid cells play essential roles in inducing protection in the host, has been described widely. Mast cells, basophils, dendritic cells, neutrophils, monocytes, and macrophages have prominent roles whereby IL-4/IL-13 polarizing cytokines participate in resolving the infection in favor of the host [20]. Basophils promote intestinal helminth clearance, inducing increased amounts of IL-4 and prostaglandins, favoring the antigen presentation to T-CD4^+^ cells, and promoting the development of M2 macrophages to repair the damaged tissues [20,21]. Another source of IL-4, essential to generating M2 macrophages during helminth infections, is the T-CD4^+^ lymphocyte, where the synergy between T cells and group 2 innate lymphoid cells (ILC2) for IL-13 production has been described [22]. IL-4 and IL-13 are necessary cytokines for switching the immunoglobulin class from IgG to IgE [23], which favors mast cell activation for histamine production [24]. However, during *T. crassiceps* infection, we observed a Th1 immune response associated with protection, where STAT1-signaling [15] and IFN-γ (unpublished data) play a significant role. Contrary to expectations, M2 macrophages and programmed cell death ligand 2 (PDL2) are essential in the susceptibility to this infection [12].

In the present work, we analyzed the role of M2 macrophages through the specific absence of IL-4Rα (cre/LoxP mice) in myeloid-derived cells, comparing our findings with the absolute lack (knockout) of IL-4Rα (IL-4Rα^−/−^). First, we found that the IL4-Rα^−/−^ mice displayed reduced parasite numbers whereas the cre/LoxP mice showed a minimum infection without signs of reduced immune response capacity; serum IgE, IL-4, and IL-13 production was increased and serum IgG1 production was raised, but IgG2a was reduced in the cre/LoxP mice, altogether indicating that IL-4Rα was functional in other cell types. However, when we analyzed cytokine production in splenocytes, we observed an increased dual Th1/Th2 profile in the cre/loxP mice to either polyclonal or antigen-specific stimulated splenocytes. This is different when we compare it with other helminth infections; for example, during schistosomiasis, IL-4/IL-13, specifically produced by Th2-CD4^+^ cells, was associated with protection. Meanwhile, Th1 response with increased TNF-α and NO production was associated with the susceptibility and death of mice at nine weeks post-infection [25]. In *Nippostrongylus brasiliensis* infection, M2 macrophages producing arginase-1, IgE-activated basophils, and IL-4 without a pro-inflammatory profile are involved in trapping larvae in the skin, avoiding lung injury [26]. These observations in schistosomiasis and *N. brasiliensis* infections are opposite to those in cysticercosis caused by *T. crassiceps* infection, where mainly a Th1-type response and M1 activation are necessary to reduce parasite load.

Our first observation analyzing the expression of M1 and M2 activation markers is that a mixed profile is present during T. crassiceps infection in the absence of IL-4 because increased expression of iNOS, Arginase, and RElm-α was observed simultaneously. Similar mixed expression was observed with macrophages stimulated with LPS/IL-4 [27]; also, it is widely recognized that helminth infections can induce a dual M1/M2 profile [28]. We also observed mixed IL-4/IFN-γ cytokine production in splenocytes from the Cre/LoxP mice. We previously observed this dual profile during colitis-associated colorectal cancer development [14]. However, we confirmed a pro-inflammatory response in cultured macrophages from *T. crassiceps*-infected cre/LoxP mice through increased NO production and reduced arginase activity similar to IL-4Rα KO mice. During M2 macrophage development, mitochondrial arginase plays a significant role in cell proliferation and collagen deposition; this role is TGF-β and IL-4/IL-13 dependent [29]. Dowling et al. recently showed that mitochondrial arginase-2 is essential for reprograming inflammatory macrophages [30]. Although *T. crassiceps* infection induced arginase activity in the WT mice, the IL-4Rα^−/−^ *T. crassiceps*-infected mice were unable to sustain arginase activity, suggesting that arginase-2 was maybe involved in the early arginase activity in these mice. However, it is necessary to analyze the activity of both mitochondrial arginases and collagen mRNA expression in cre/loxP mice to understand its role in cysticercosis. The expression of M2-associated molecules in the absence of IL-4Rα signaling was an unexpected result. However, it is widely accepted that IL-4Rα is involved in type I and type II IL-4 signaling; type I requires the γ common chain, and type II requires the IL-13Rα1 chain for STAT6-mediated signaling phosphorylation [31]. The presence of M2 markers in the absence of IL-4 signaling could suggest a role for specific IL-13Rα2, where this receptor induces the phosphorylation of the STAT6 transcription factor. There is evidence supporting this hypothesis [32]. Also, IL-13 through the IL-13Rα2 receptor induces increased TGF-β expression, but collagen and TGF-β1 are reduced through IL-13Rα2 silencing [33]. Overexpression of IL-13Rα2 has been described in oncological pathologies like glioblastoma, adrenocortical carcinoma, head and neck cancer, melanoma, ovarian cancer, and pancreatic cancer [34], where anti-inflammatory and immunosuppressor environments are necessary for tumor survival; the M2-Th2 microenvironment in oncological pathologies is similar to that observed in helminth infections. Our data support the hypothesis that, in the absence of IL-4Rα type II and type II signaling, IL-13Rα2 could probably even play a major role in inducing compensatory mechanisms involved in the susceptibility and resistance to *T. crassiceps* infection. However, this needs to be addressed in future experiments.

Flow cytometry analyses of myeloid cells showed the recruitment at the site of infection of Ly6G^+^, Ly6C^hi^, and Ly6C^low^ cells in the absence of IL-4Rα. We recently found that the recruitment of myeloid cells during *T. crassiceps* infection is a STAT-1-dependent event [15]. Thus, for protection during *T. crassiceps* infection, a pro-inflammatory profile of monocytes and neutrophils is required. Interestingly, cre/LoxP mice did not show an increased percentage of these cells. Probably, the absence of IL-4 signaling, specifically in myeloid cells, plays a significant role in the expression of chemokine receptors needed for the recruitment of myeloid cells; IL-4 is necessary for both αvβ3 [35,36] and β5 integrins’ expression on immune cells, with this being of relevance for the establishment of myeloid cells at the site of infection [37]. On the other hand, the pro-inflammatory M1 macrophage profile has been associated with protection during intracellular protozoan [38] and bacterial infections [39]. As we described, in *T. crassiceps* infection, protection is associated with a pro-inflammatory profile in macrophages, and susceptibility is M2 dependent [12]. Here, M1 macrophages showed an increased percentage of MHC-II expression in all experimental groups. However, M2 macrophages showed increased expression of PD-L2 only in the *T. crassiceps*-infected WT mice and reduced expression in the IL-4Rα^−/−^ mice. PD-L2 has an inducible expression in macrophages, dendritic cells, and mast cells. Its role is involved in maintaining peripheral tolerance and the stability of T cells [40] and inducing anergy or apoptosis in immune active cells [41]. According to our results, macrophages from *T. crassiceps*-infected cre/loxP mice lack PD-L2 expression; this may favor the appropriate function of effector cells like CD8^+^ and NK cells, which are IFN-γ producers and may maintain M1 activation through NO production, reducing the parasite load [42]. It has been described that the persistence of human papillomaviruses is associated with increased myeloid cells expressing PD-L1 and PD-L2 and decreased circulating NK cells [43]. During *Salmonella typhimurium* infection, infected cells evade T-CD8 response by increasing the expression of PD-L1 and PD-L2 molecules in B cells and macrophages [44]. Interestingly, the low expression of PD-L2 in macrophages from *T. crassiceps*-infected cre/LoxP mice, the reduced expression of PD-1 and Tim-3 in CD8^+^ cells, but the increased expression of the CD25 activation molecule suggest a restoration in the effector profile of CD8 cells. It is necessary to analyze if NK cells have a restoration in their effector profile and if they have a putative role in cysticercosis protection.

Finally, the role of T cells during helminth infections has been widely reported, mainly due to its IL-4 production in a STAT6-dependent fashion, inducing the anti-inflammatory profile needed to generate protection [45]. During *T. crassiceps* infection, we previously reported susceptibility, but no resistance is STAT6 mediated [46]. Interestingly, our new results suggest that the resistance to cysticercosis observed in cre/loxP mice is mediated by the absence of suppressive M2 response, which is IL-4Rα dependent. However, the pro-inflammatory response remains efficient with increased IFN-γ, IL-6, TNF-α production, and M1 activation.
